# Probiotic prophylaxis to prevent ventilator-associated pneumonia in children on mechanical ventilation: A randomized double-blind clinical trial

**DOI:** 10.3389/fped.2022.1045941

**Published:** 2022-11-15

**Authors:** Soheil Roshanzamiri, Maryam Alemzadeh, Seyyedeh Narjes Ahmadizadeh, Azita Behzad, Seyyedeh Masumeh Hashemi, Jamshid Salamzadeh, Bahador Mirrahimi

**Affiliations:** ^1^ Department of Clinical Pharmacy, School of Pharmacy, Shahid Beheshti University of Medical Sciences, Tehran, Iran; ^2^ Department of Pediatric Intensive Care, Mofid Children Hospital, Shahid Beheshti University of Medical Sciences, Tehran, Iran

**Keywords:** probiotics, ventilator-associated pneumonia (VAP), intensive care units, pediatric, mechanical ventilation

## Abstract

**Purpose:**

Ventilator-Associated Pneumonia (VAP) is one of the most common nosocomial infections in the Pediatric Intensive Care Unit (PICU). Using new strategies to prevent nosocomial infections is crucial to avoid antibiotic resistance. One of these strategies is the utilization of probiotics. This study aims to investigate the efficacy of probiotic prophylaxis in preventing VAP in mechanically ventilated children.

**Method:**

This study was a randomized, double-blind clinical trial. The study included 72 children under 12 years of age under mechanical ventilation for more than 48 h in the Mofid Children's Hospital. Patients were randomly divided into Limosilactobacillus reuteri *DSM 17938* probiotic recipients (*n* = 38) and placebo groups (*n* = 34). In addition to the standard treatment, both groups received a sachet containing probiotics or a placebo twice a day. Children were screened for VAP based on clinical and laboratory evidence.

**Results:**

The mean age of children in the intervention and placebo groups was 4.60 ± 4.84 and 3.38 ± 3.49 years, respectively. After adjusting the other variables, it was observed that chance of VAP among probiotics compared to the placebo group was significantly decreased (OR adjusted = 0.29; 95% CI: 0.09–0.95). Also, probiotic was associated with a significantly lower chance of diarrhea than the placebo group (OR adjusted = 0.09; 95% CI: 0.01–0.96).

**Conclusion:**

Probiotic utilization is effective in preventing the incidence of VAP and diarrhea in children under mechanical ventilation in the PICU.

## Introduction

Hospital-related diseases are among the significant problems in the Pediatric Intensive Care Unit (PICU) (1). Nosocomial infections increase morbidity, mortality, and the duration of hospitalization (2). Ventilator-Associated Pneumonia (VAP) is one of the most common infections in the PICU; the cumulative incidence of VAP in patients under mechanical ventilation is 22.8% (3). VAP leads to increased hospitalization, Intensive Care Unit (ICU) stay, and costs imposed on patients and the health care system (4).

According to a recent study, the prevalence of nosocomial infections in Iran was 4.5%. VAP and hospital-acquired pneumonia (HAP) are among the common nosocomial infections in this country. The prevalence of pneumonia may rise due to the installed ventilation systems in many developing countries with insufficient facilities, such as Iran (5, 6).

In recent years, studies have investigated various methods of VAP prevention. The administration of probiotics is one of the latest strategies leading to VAP prevention with various local and systemic mechanisms. These mechanisms modulate the host defense response or reduction of colonization (7). Various studies have shown the positive effect of probiotics in preventing VAP (8–11). However, some studies reported that the administration enteral of probiotics, compared with placebo among critically ill patients requiring mechanical ventilation, has no statistically significant impact on the development of VAP (9, 12–15).

It should be noted that probiotics are live microorganisms, so the risk of iatrogenic infections increases with their administration (15, 16). A recent meta-analysis showed a positive effect of probiotics in mechanically ventilated patients. However, it emphasized the importance of assessing the efficacy and safety of probiotics in critically ill children (17). In addition to the high prevalence and mortality rate of VAP in the PICU, there are few studies on the preventative role of probiotics in children. This study aimed to determine if *Limosilactobacillus reuteri DSM 17938* (formerly known as *Lactobacillus Reuteri DSM 17938*) decreases VAP and other clinically meaningful outcomes compared to a placebo in critically ill children undergoing mechanical ventilation.

## Materials and methods

This double-blind, placebo-controlled clinical trial was done in a PICU at a university-affiliated tertiary care teaching hospital (Mofid Children Hospital, Tehran, Iran) from May 2021 to July 2022. The Research Ethics Committees of Shahid Beheshti University of Medical Sciences approved the study (IR.SBMU.PHARMACY.REC.1399.383). Additionally, the trial was registered with the Iranian Registry of Clinical Trials (IRCT20120415009475N9).

Before including the subjects in the study, written informed consent was obtained from their parents. Patients who were under mechanical ventilation for over 72 h at the time of screening, children at potential increased risk of iatrogenic probiotic infections (HIV infection, malignancy requiring chemotherapy, previous transplantation, and those receiving chronic immunosuppressive such as chemotherapy agents and immunosuppressive medications, and high dose corticosteroids (more than 1 milligram per kilogram), children with paralytic ileus, inability to receive enteral medications, COVID-19 infection (due to effect of this disease on the lung) and children with gastrointestinal bleeding were excluded from the study. All children aged 28 days to 12 years who required mechanical ventilation for more than 48 h were recruited.

### Interventions

Children assigned to the intervention group received a probiotic sachet containing 8 × 10^8^ colony-forming units of *Limosilactobacillus reuteri DSM 17938* (Farabiotic Pharmaceuticals, Tehran, Iran) in a sachet suspended in 5 ml of water and *via* a nasogastric tube twice daily for 7 days or until discharged from PICU, whichever comes first. The initial dose was administered within 48 h following intubation. The placebo group got a sachet containing maltodextrin (Farabiotic Pharmaceuticals, Tehran, Iran) identical to the *Limosilactobacillus reuteri DSM 17938* sachet for 7 days or till discharge. Similarly, the placebo was dissolved in water and administered twice daily. Placebo has the same appearance and consistency as the probiotic when suspended in water. The placebo was prepared by the same company that produced *Limosilactobacillus reuteri DSM 17938*.

### Data gathering

The clinical pharmacy resident and PICU fellow reviewed patients daily under supervision. During these visits, demographic information, the reason for hospitalization and intubation, Pediatric Index of Mortality 3 (PIM-3) calculation (18), culture results, clinical diagnoses, diarrhea episodes, duration of mechanical ventilation, ICU and hospital stay, and ICU mortality were collected using a research-made checklist.

#### Study definitions

VAP develops at least 48 h after endotracheal intubation, whereas it was not present when mechanical ventilation was initiated. Diagnosis of VAP events was complex and controversial, and the inclusion of subjective clinical signs and symptoms may contribute to variations in reported VAP rates. Due to the high diagnostic value of Mini bronchoalveolar lavage (mini-BAL) (as the gold standard diagnostic test), only cases with clinical symptoms or radiographic changes and a positive culture were confirmed as definitive VAP. Diarrhea in children was also investigated. This study defines diarrhea as the passage of three loose or watery stools within 24 h.

#### Outcomes and follow-up

The primary outcome was the incidence of VAP. Incidence of diarrhea, the length of mechanical ventilation, ICU and hospital stay, and ICU mortality were secondary outcomes. In situ devices like central venous catheters and urine catheters, vasopressor administration, bacterial colonization, and prokinetic use were compared between the two groups.

Patients who enrolled in the study were assessed daily for clinical signs of VAP. Mini-BAL, complete blood count, and blood cultures were sent anytime there was clinical suspicion of VAP. Patients with VAP were treated following the standard guidelines. Patients were monitored for incidence of VAP for 14 days after receiving the probiotic.

#### Sample size estimation

Based on the previous study, the incidence of VAP in the placebo group was 40%, and in the intervention group was 19.1% (19). A total of 83 participants in each group were estimated at 5% significance (*α* = 0.05) with 80% power (*β* = 0.2). By considering a 15% probability of attrition rate, about 100 subjects in each group were considered (*N*_total_ = 200).

#### Interim analysis

**Figure 1:**
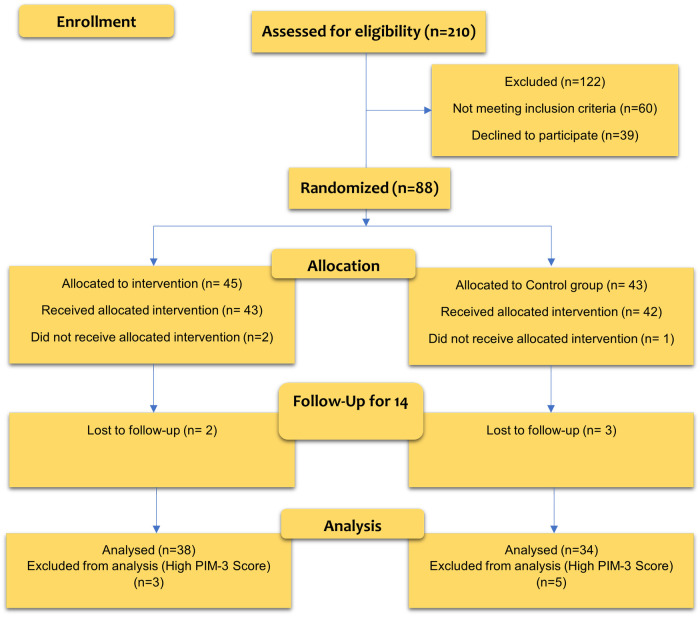
CONSORT diagram of the study.

Considering that more than 25% of the total patients were included in the study, an interim analysis was performed. In this interim analysis, it was found that probiotic prophylaxis effectively prevents VAP, and the study was terminated due to the benefits of patients receiving a placebo. Finally, the placebo and intervention groups’ sample sizes were 34 and 38, respectively. Figure 1 shows the CONSORT diagram of the study.

#### Randomization

All children admitted to PICU were screened during the study period, and those who satisfied the inclusion criteria were recruited. Randomization has been done using block randomization. Patients were randomly allocated in 1:1 to placebo or intervention groups. Our study randomized samples in groups of 4 patients (50 blocks). Pooled and computerized randomization with secret blinding is performed on a site https://www.sealedenvelope.com/. The probiotics and placebo were in look-alike non-consecutive coded packages. The codes based on the block randomization table were provided to the researcher by a designated person *via* phone. At the end of the study, the statistical expert performed the analysis after organizing the data by the same person. The code packet was then opened, and the results were reported.

### Blinding

This study was a double-blind, placebo-controlled clinical trial that all potential participants (patient, family, physicians, clinical pharmacist) were blinded to treatment allocation to reduce selection bias.

#### Statistical analysis

The statistical analysis was conducted at a significant level of less than 0.05 with a 95% Confidence Interval (CI). The analysis was performed using STATA software version 14.

The continuous variables’ normality was assessed using the Shapiro-Wilk test and Q-Q plot. For the description of continuous variables, mean and Standard Deviation (SD) and categorical variables were used from frequency and percentage (%). Due to the non-normally distribution of continuous variables, the Mann-Whitney *U* test was used to compare the value of these variables between groups.

In this study, survival probability was reported using the Kaplan-Meier method, and the *p*-value for comparison of survival probability between two groups was estimated using the log-rank test.

Univariate and multivariable regression models were used to identify the association between under independent factors and outcomes. A backward stepwise approach with *p*-value <0.2 was used to select the best variables to enter the last multivariable model. In addition, some variables that did not have statistical criteria for entering the multivariable model (*p*-value >0.2) due to approved clinical effects and the potential role of residual confounding of these variables were adjusted in the last multivariable model. Logistic regression was used to assess the association between outcomes (diarrhea and VAP) and selected variables at univariate and multivariable levels. Finally, the best multivariable logistic regression model was fitted based on the value of Area Under Curve (AUC, ROC curve) and the Hosmer-Lemeshow test.

## Results

Baseline characteristics of both probiotics and placebo groups were comparable, and there was no significant difference between any parameter in the two groups (*p* > 0.05) (Table 1). The mean age of children in the probiotics group was 4.60 ± 4.84 years, and in the placebo group was 3.38 ± 3.49 years, which was not statistically significant (*p* = 0.487).

**Table 1:** Baseline characteristics between intervention and placebo groups.

Variables	All patients (*n* = 72)	Placebo (*n* = 34)	Intervention (*n* = 38)	*p*-value
General information				
Age (in years)	4.02 ± 4.27	3.38 ± 3.49	4.60 ± 4.84	0.487
Age (≤1 year)	28 (38.89)	13 (38.24)	15 (39.47)	0.914
Age (>1 year)	44 (61.11)	21 (61.76)	23 (60.53)	0.598
Gender/Male	40 (55.56%)	20 (58.82%)	20 (52.63%)	
Gender/ Female	32 (44.44%)	14 (41.18%)	18 (47.37%)	
Weight (in kg)	14.70 ± 12.34	13.41 ± 10.08	15.87 ± 14.10	0.973
PIM3 Score (Percent)	4.25 ± 4.22	4.87 ± 4.82	3.71 ± 3.59	0.360
Hospital admission causes				
Cardiovascular disease	1 (1.39)	0 (0.00)	1 (2.63)	0.047*
Foreign body	5 (6.94)	5 (14.71)	0 (0.00)	
Infectious diseases	3 (4.17)	0 (0.00)	3 (7.89)	
Intoxication	2 (2.78)	0 (0.00)	2 (5.26)	
Metabolic disease	4 (5.56)	1 (2.94)	3 (7.89)	
Neurological disease	41 (56.94)	20 (58.82)	21 (55.26)	
Surgery	12 (16.67)	7 (20.59)	5 (13.16)	
Traumatic injury	4 (5.56)	1 (2.94)	3 (7.89)	
Ventilation causes				
Cardiac arrest	2 (2.78)	0 (0.00)	2 (5.26)	0.631
Loss of consciousness	7 (9.72)	3 (8.82)	4 (10.53)	
Post-operation	14 (19.44)	8 (23.53)	6 (15.79)	
Respiratory failure	49 (68.06)	23 (67.65)	26 (68.42)	

PIM3, pediatric index of mortality 3. Values are *n* (%) or mean ± SD.

* Statistically significant, *p*-value <0.05.

The types of organisms from mini-BAL showed in Table 2. Klebsiella (29.17%), Pseudomonas aeruginosa (25%) and Acinetobacter (16.67%) were the most frequent bacteria among children with positive microbiology tests. There was no difference in the distribution of pathogen types between the two groups (*p* = 0.442).

**Table 2:** Types of organisms from mini-BAL.

Variables	Total positive test (*n* = 24)	Placebo (*n* = 15)	Intervention (*n* = 9)	*p*-value
Pathogens				
*Acinetobacter*	4 (16.67)	4 (26.67)	0 (0.0)	0.442
*Escherichia coli (E. coli)*	2 (8.33)	1 (6.67)	1 (11.11)	
*Haemophilus*	1 (4.17)	1 (6.67)	0 (0.0)	
*Klebsiella*	7 (29.17)	3 (20.00)	4 (44.44)	
*Pseudomonas aeruginosa*	6 (25.00)	4 (26.67)	2 (22.22)	
*Staphylococcus aureus*	3 (12.50)	1 (6.67)	2 (22.22)	
*Streptococcus*	1 (4.17)	1 (6.67)	0 (0.0)	

Values are *n* (%).

Although there was a statistically significant difference between the placebo and probiotic groups in the hospital admission causes (*p* = 0.047), there was no association between this factor and outcomes (*p*-value greater than 0.05).

The incidence of VAP was significantly lower in the probiotics group compared to the placebo group. Eight pediatric patients in the probiotics group (21.05%) had VAP, compared to 15 children in the placebo group (44.12%) (*p* = 0.036) (Table 3). Administration of probiotics was associated with a significantly lower chance of diarrhea than the placebo group (2.63% vs. 20.59%; *p* = 0.023. The placebo group had higher colonization rates than the probiotics group (70.59% vs. 44.74%; *p* = 0.027).

**Table 3:** Outcome variables.

Variables	All patients (*n* = 72)	Placebo (*n* = 34)	Intervention (*n* = 38)	*p*-value
Outcomes				
Mortality in ICU (Yes)	11 (15.28%)	7 (20.59%)	4 (10.53%)	0.641
VAP Incidence (Yes)	23 (31.94%)	15 (44.12%)	8 (21.05%)	0.036*
Diarrhea (Yes)	8 (11.11%)	7 (20.59%)	1 (2.63%)	0.023*
Ventilation time (h)	242.30 ± 164.76	268.82 ± 175.68	218.57 ± 152.77	0.238
ICU stay (days)	15.11 ± 12.15	15.88 ± 14.72	14.42 ± 9.43	0.896
Hospital stays (days)	29.12 ± 21.53	31.96 ± 22.46	26.46 ± 20.62	0.290
Bacterial Colonization (Yes)	41 (56.94%)	24 (70.59%)	17 (44.74%)	0.027*

ICU, intensive care unit; VAP, ventilator-associated pneumonia. Values are *n* (%) or mean ± SD.

* Statistically significant, *p*-value <0.05.

**Figure 2:**
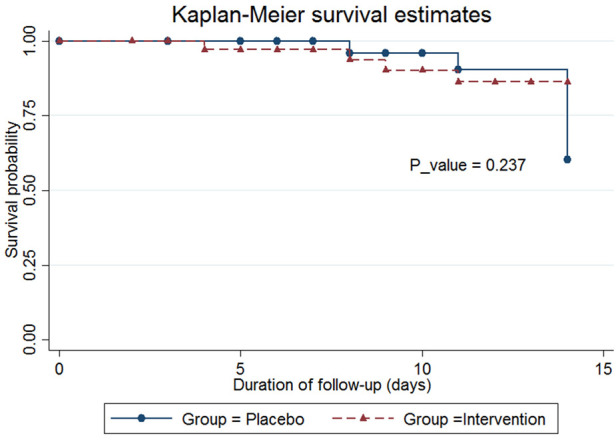
Kaplan-Meier analysis of ICU mortality.

The mean length of ICU stay in the probiotics group was 14.42 ± 9.43 days, compared to 15.88 ± 14.72 days in the placebo group (*p* = 0.896); however, this difference was not statistically significant. The mean duration of hospitalization in the probiotics group was 26.46 ± 20.62 days, compared to 31.96 ± 22.46 days in the placebo group (*p* = 0.290). The mean ventilation duration in the probiotics group was 218.57 ± 152.77 h, compared to 268.82 ± 175.68 h in the placebo group (*p* = 0.238). The occurrence of 14-day mortality and the probability of survival was not statistically significant in the placebo group compared to the probiotic group (20.59% vs. 10.53% *p* = 0.641) (Table 3, Figure 2).

The results of the study that evaluated the risk factors for nosocomial infections between the two groups are shown in Table 4. Multivariable logistic regression analysis was used to minimize the effect of these confounding variables. Based on the multivariable logistic regression model, compared to the placebo group, the probiotic group was 70% less likely to have VAP (OR_adjusted_ = 0.29; 95% CI 0.09–0.95) (Tables 2, 5). The chance of diarrhea was reduced by about 91% in the probiotic group compared to placebo (OR_adjusted_ = 0.09; 95% CI 0.01–0.96) (Tables 2, 6). No adverse reactions were reported during this study.

**Table 4:** Risk factors for nosocomial infections.

Variables	All patients (*n* = 72)	Placebo (*n* = 34)	Intervention (*n* = 38)	*p*-value
Risk factors				
Multi drug resistance pathogen (Yes)	21 (29.17)	12 (35.29)	9 (23.68)	0.279
Vasopressor administration (Yes)	38 (52.78)	18 (52.94)	20 (52.63)	0.979
Antibiotics prior to admission (Yes)	3 (4.17)	1 (2.94)	2 (5.26)	1.000
Urinary catheterization (Yes)	68 (94.44)	33 (97.06)	35 (92.11)	0.617
Central venous catheter (Yes)	63 (87.50)	30 (88.24)	33 (86.84)	1.000
Parenteral nutrition (Yes)	2 (2.78)	1 (2.94)	1 (2.94)	1.000

Values are *n* (%).

**Table 5:** Factors associated with VAP based on univariate and multivariable logistic regression model.

Variables	Crude OR^a^, 95% CI	*p*-value	Adjusted OR, 95% CI	*p*-value
Age (≤1 year)	Reference	0.289	Reference	0.454
Age (>1 year)	0.57 (0.21–1.58)		0.65 (0.21–2.006)	
Gender/Male	Reference	0.105	Reference	0.205
Gender/Female	0.42 (0.14–1.20)		0.47 (0.14–1.50)	
PIM3	1.02 (0.91–1.15)	0.647	0.97 (0.85–1.11)	0.733
Group				
Placebo	Reference	0.039*	Reference	0.041*
Intervention	0.33 (0.12–0.94)		0.29 (0.09–0.95)	
ICU stays (days)	1.03 (0.99–1.08)	0.115	1.04 (0.96–1.12)	0.277
Ventilation time (h)	1.001 (0.99–1.004)	0.272	0.99 (0.99–1.004)	0.874
Vasopressor administration/No	Reference	0.055	Reference	0.091
Vasopressor administration/ Yes	2.80 (0.98–8.02)		2.72 (0.85–8.68)	

ICU, intensive care unit; PIM3, pediatric index of mortality 3.

* Statistically significant, *p*-value <0.05.

^a^ Odds ratio, 95% confidence interval.

**Table 6:** Factors associated with diarrhea based on univariate and multivariable logistic regression model.

Variables	Crude OR^a^, 95% CI	*p*-value	Adjusted OR, 95% CI	*p*-value
Age (≤1 year)	Reference	0.400	Reference	0.179
Age (>1 year)	2.05 (0.38–10.97)		4.05 (0.52–31.21)	
Gender/Male	Reference	0.676	Reference	0.931
Gender/Female	0.72 (0.15–3.28)		0.92 (0.15–5.58)	
Group				
Group/Placebo	Reference	0.040*	Reference	0.046*
Group/Intervention	0.10 (0.01–0.89)		0.09 (0.01–0.96)	
ICU stays (days)	1.03 (0.98–1.08)	0.162	1.02 (0.97–1.08)	0.317
MDR pathogens/ Negative	Reference	0.040*	Reference	0.135
MDR pathogens/ Positive	5.00 (1.07–23.30)		3.86 (0.65–22.84)	
Vasopressor administration/No	Reference	0.198	Reference	0.260
Vasopressor administration/Yes	3.00 (0.56–15.99)		2.95 (0.44–19.42)	

ICU, intensive care unit; MDR, multi-drug resistant.

* Statistically significant, *p*-value <0.05.

^a^ Odds ratio, 95% confidence interval.

## Discussion

The primary goal of this study was to investigate the effect of probiotics on the incidence of VAP in critically ill pediatric patients considering the lack of studies in this area (20–22). Our study is the first randomized, double-blind, placebo-controlled trial in critically ill children aged from 28 days to 12 years with VAP as the primary outcome. The effectiveness of *Limosilactobacillus reuteri DSM 17938* in preventing VAP was first examined in our study. Other studies have used *Lactobacillus rhamnosus GG* or a combination of several probiotics (7, 15, 19). The present study is distinctive because it confirmed the microbiologic diagnosis of VAP using lower respiratory tract sampling and quantitative cultures (mini-BAL).

The primary cause of VAP is the aspiration of colonized pathogenic bacteria into the oropharynx and gastrointestinal tract. The most critical risk factors for VAP are intubation and the length of mechanical ventilation (23). Through various local and systemic actions that minimize the colonization of more virulent species or boost host immune systems, probiotics may effectively reduce the incidence of VAP. These consequences include a reduction in the colonization of pathogenic microorganisms, an alteration in upper respiratory tract flora, enhancement in the function of the gut mucosal barrier, reduction in bacterial translocation, and modulation of the immune system (24, 25). Similar to previous studies, our research found that bacterial colonization was lower in probiotic groups than in placebo groups (21, 26, 27).

The administration of probiotics in the intervention group significantly reduced the incidence of VAP in children undergoing mechanical ventilation; however, preliminary analysis revealed that the intervention did not associate with decreasing ICU stay or mortality. Similar to our study, several other clinical trials, both in children and adults, reported a decrease in the incidence of VAP in the probiotic group (21, 22, 28). In contrast, some studies failed to demonstrate that probiotics have a preventive effect against VAP (15, 20, 29).

In a study conducted in 2017 at the same hospital, the incidence of VAP in children undergoing mechanical ventilation was 21% (30), which is substantially lower than the risk found in this research and other comparable studies (3, 22, 26). In our study, the incidence of VAP in the probiotics group was significantly lower than in the placebo group; 21.05% vs. 44.12%, respectively (*p* = 0.036). The efficiency of probiotics in preventing VAP in critically ill patients has been challenged in recent years due to many reasons, including population differences in each study, challenges in the definition and diagnosis of VAP, and differentiation from noninfectious causes, type of probiotic, dosage, and duration of administration (26). However, the most recent meta-analysis has demonstrated probiotics efficacy; study of 23 trials with a total of 5,543 individuals, the relative risk pooled estimation of probiotics in reducing the incidence of VAP in neonates and children was 0.55 (*n* = 407; 95% CI = 0.31 to 0.99; *p* = 0.046) that was statistically significant.

Diarrhea is one of the most worrisome ICU complications and may contribute to the increasing hospital and ICU stays and mortality rates (31, 32). The incidence of diarrhea in ICUs varies widely between studies, ranging from 9.7% to 41% (32). However, limited studies have been conducted on diarrhea's incidence, mechanism, and etiology in critically ill children and adults. The possible mechanisms of diarrhea in the ICUs include alteration in the gut microbiota during hospitalization, broad-spectrum antibiotics, laxative medications, pancreatic exocrine failure, and enteral Feeding (33). In this study, children treated with probiotics experienced a statistically significant reduction in the incidence of diarrhea than those treated with the placebo. Similarly, probiotics can decrease the incidence of diarrhea in critically ill patients (34), but their mechanisms are not yet fully understood; however they are now thought to work primarily by preventing disturbances of the cytoskeleton and tight junctions, altering host defenses, inducing the production of IgA, and competing for adhesion sites (35). The efficacy of *Limosilactobacillus reuteri DSM 17938* in preventing diarrhea in critically ill hospitalized children has not yet been investigated.

Secondary outcomes are all shown in Table 3. Probiotic administration did not decrease the duration of ventilation significantly. ICU and hospital length of stay data did not differ significantly between treatment and placebo groups; the 14-day mortality rate and the probability of survival were not statistically significant in the placebo compared to the probiotic group. These results were consistent with the other studies (19, 36). In general, the probiotic group had shorter hospital and ICU stays, less mechanical ventilation time, and lower mortality rates; Nevertheless, these differences were not statistically significant, but the reduction of ICU mortality is very promising and valuable for further studies.

In this trial, probiotics did not show any harmful effects. *Limosilactobacillus reuteri DSM 17938* is a gram-positive bacterium that belongs to the larger group of lactic acid bacteria. *Limosilactobacillus reuteri DSM 17938* is generally regarded as safe (GRAS). Lactobacilli and other probiotics do not usually colonize the gastrointestinal system since they are undetectable a few days after treatment cessation (37). Generally, infections caused by probiotics containing Lactobacilli microorganisms are uncommon in the human population. This lack of pathogenicity applies to all age groups and immunocompromised people (38). However, newborns and immunocompromised children were excluded from the study as a measure of extra caution.

The probiotic agent chosen for this investigation (*Limosilactobacillus reuteri DSM 17938*) was distinct from the agent(s) utilized in previous studies. However, due to a lack of comparable evidence in this area, it remains unclear if this probiotic is more beneficial than other probiotics for preventing VAP.

This study used maltodextrin as a placebo and excipient (800 mg in the placebo sachet and 500 mg in the probiotic sachet). Although maltodextrin has prebiotic effects and could act as a confounding factor, a review of the literature revealed that the prebiotic effects of maltodextrin were dose-dependent, and different studies used doses of more than 5,000 mg (39).

This research has a variety of limitations: (1) These data were collected from a single facility and are subject to inherent biases. (2) It was conducted in a single ICU where most patients were surgical cases requiring less than 48 h of ventilation. (3) This hospital is a referral center, and many patients were transferred to this ICU from other hospitals; thus, many patients were admitted to the ICU for longer than 72 h after beginning mechanical ventilation and excluded from the study. (4) Due to the outbreak of Delta and Omicron peak of Covid-19 and the fact that the ICU was full of children infected with this disease, it was challenging to continue the research for many months.

To our knowledge, this study's first double-blind methodology in critically ill children and confirmation of VAP based on microbiological criteria reduced the reporting of tracheal colonization and ventilator-associated tracheobronchitis as VAP.

Due to the increasing prevalence of nosocomial infections caused by MDR pathogens, it is crucial to implement additional strategies to decrease hospital-acquired infections. Probiotics appear safe and are associated with a significant decrease in the incidence of VAP, bacterial colonization, and diarrhea. Still, the length of hospital stay in the probiotic group was lower than in the placebo group; Although, this difference was not statistically significant.

## Data Availability

The original contributions presented in the study are included in the article/Supplementary Material, further inquiries can be directed to the corresponding author/s.
